# Intravitreal dexamethasone: variation of surgical technique and prevention of ocular complications with ASOCT follow-up

**DOI:** 10.1007/s00417-022-05650-5

**Published:** 2022-04-26

**Authors:** Raffaele Nuzzi, Alessandro Rossi, Alessia Nuzzi

**Affiliations:** 1 Institute of Ophthalmology, Department of Surgical Sciences, University of Turin, Via Cherasco 23, 10126 Turin, Italy; 2 Department of Clinical Sciences and Community Health, Eye Clinic San Giuseppe Hospital, IRCCS Multimedica, University of Milan, Milan, Italy

**Keywords:** Anterior segment OCT (ASOCT), Ozurdex, Intravitreal treatment, Dexamethasone, Maculopathy

## Abstract

**Purpose:**

To verify the correct decision-making procedure on performing an intravitreal injection by investigating the in vivo wound morphology and evolution of 22-gauge wounds after dexamethasone oblique injection with anterior segment optical coherence tomography (OCT).

**Design:**

Prospective, observational consecutive case series.

**Methods:**

Subjects underwent a dexamethasone injection at University Eye Clinic of Turin. All the injections have been performed in an oblique (aka beveled or angled) fashion. Patients were divided according to the number of injections already performed with dexamethasone. Group 1 consisted of patients at the first injection, group 2 of patients at a second or more injection always in the same quadrant, and group 3 of patients at the second or more injection in a different quadrant. The incisions were imaged with the Heidelberg SPECTRALIS OCT device on postoperative days 1, 8, and 15. The main outcome measure was wound structure/characteristics (e.g., presence of gaping) as evaluated with OCT. Surgical and ocular parameters were also recorded.

**Results:**

Thirty-three consecutive patients were investigated. OCT demonstrated closed wounds in all eyes on postoperative days 1, 8, and 15. In all patients, the external (entry) side of the incision was seen as a gape; however, the rest of the wound was closed. No complications were recorded in the different patients during the follow-up. In patients of group 1, we identified the scleral pathway in 10 eyes at day 1. At 8 days in 9 of 10 eyes, the sclera had returned to its *restitutio ad integrum*. In patients of group 2, the scleral pathway was recognizable on the first day of control; in 7 patients, this was accompanied by the presence of intrascleral edema with peri-wound fluid. At the 8-day checkup, 3 eyes still showed signs attributable to the intrascleral pathway accompanied by peri-wound edema. In group 3, it was possible to identify the intrascleral pathway in 8 patients. There were no signs of intrascleral peri-wound edema or other anatomical changes in 9 patients as early as the first day. In the 8-day follow-up, the signs of scleral edema were absent in the single patient who presented them. At 15 days, there were no signs of scleral pathway in all eyes analyzed.

**Conclusions:**

Speaking of intravitreal injections of slow-release dexamethasone, the technique that involves moving the conjunctiva and a beveled or angled sclerotomy after a careful choice of the injection site, paying attention to vary the quadrant involved with each puncture, reduces the number of days of closure of the sclera via and the scleral damage, thus protecting the patient from complications. For the future, it is hoped that the operating microscope and intraoperative OCT will be used on every occasion.


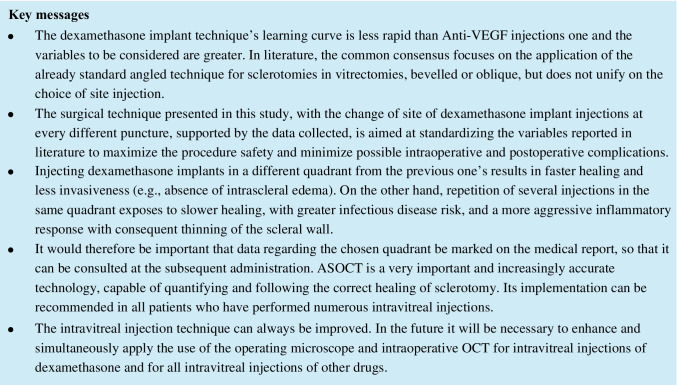


## Introduction

Ozurdex (Allergan Inc., Irvine, CA, USA) is a biodegradable, slow-release dexamethasone implant delivered by intravitreal injection. It has been studied mainly for the treatment of macular edema (ME) associated with retinal vascular occlusion (RVO) [1] and non-infectious posterior uveitis, and has been approved by the regulatory agencies in the USA and Europe for these indications [2][2][2]. Dexamethasone was also shown to be effective in the treatment of diabetic macular edema (DME) [5][5], and several other studies have reported its efficacy in treating ME secondary to numerous other diseases, including Irvine-Gass syndrome [7], Coat’s disease [8], radiation retinopathy [9], cystoid macular edema (CME) secondary to retinitis pigmentosa [9], and acute optic neuritis, particularly in the acute phase of the disease [10].

Despite the widespread use of this drug today, few studies evaluate the accuracy of the surgical approach, the correct realization of the different steps, and the details of the procedure itself.

For the intravitreal injection technique of drugs in vials (anti-VEGF), the learning curve of operators is very rapid, and the risks associated with the surgical procedure are minimal [11]. For the dexamethasone implant, given the width of the needle and the scleral trauma exerted by the implant device, the learning curve is less rapid and the variables to be considered are greater [12]. In literature, the common consensus focuses on the application of the already standard angled technique for sclerotomies in vitrectomies [13], beveled or oblique, but does not unify on the choice of site injection.

The surgical technique presented in this study and supported by the data collected is aimed at standardizing the variables reported in literature to maximize the procedure safety and minimize possible intraoperative and postoperative complications. The aid of the OCT of the anterior segment (ASOCT), as a device for verifying the rapid and correct healing of the entry site, also supports the preservation of the anatomical structures and the minimum trauma exercised.

## Methods

### Patients

We prospectively evaluated 33 consecutive patients who met the following inclusion criteria: (1) were clinical candidates to perform an intravitreal injection of dexamethasone for RVO or DME; (2) willing and able to give written informed consent. Patients with a history of prior scleral buckling or pars plana vitrectomy (PPV) were excluded. Patients underwent ASOCT imaging on postoperative days 1, 8, and 15. All operations were performed at the University Eye Clinic of Turin from May 1, 2021, to May 30, 2021, by one surgeon (R.N.). Patient medical records were reviewed, and surgical and ocular parameters were recorded.

The patients were divided into three groups. Group 1 consisted of patients at first injection (14), the second were patients at the second or more injection who underwent the procedure in the same quadrant as the previous times (9), and the third were patients at the second or more injection who performed the puncture in a different quadrant than the previous punctures. The subdivision into three groups was purely observational. The injection technique chosen was identical for all patients (patient demographic data are summarized in Table 1).

**Table 1:** Patient demographic data

	Group 1	Group 2	Group 3
No. of patients	14	9	10
Age	75	70	72
Male	8	4	5
Right eye	6	5	5
Mean IOP pre	15	14	16
DME	10	6	7
BRVO/CRVO	4	3	3

The operator in charge of performing the injection was not aware of the number of punctures already performed by the patient and performed the puncture in a location previously indicated in the medical record. The injection quadrant was chosen randomly for the first injections or in any case randomly from those not yet used previously. The operator who performed the OCT exams did not know the number of injections performed and how many of these had been performed in the quadrant he was going to analyze. The data were subsequently crossed by a third operator.

In the selected patients, only 5 already had ocular pathologies: two patients in group 1 and one patient in group 2 had a history of developed uveitis; all of them had previously open-angle glaucoma (POAG), as well as two other patients, with no uveitis, in group 3.

### Surgical procedure

All patients received a single eye injection of Ozurdex with the dexamethasone injector. The surgical technique involves the following steps:

1. Preparation: 2 drops of oxibuprocaine hydrochloride single-dose eye drops were instilled three times at 10 min; then, bulb disinfection was done upon entering the operating room with an antibiotic-cortisone combination (dexamethasone + netilmicin) followed by a drop of povidone-iodine eye drops (0.5%). After this step, periocular skin was carefully disinfected using a povidone-iodine solution (7.5%), followed by the placement of disposable blepharostat and another bulb disinfection by alternating a drop of iodopovidone eye drops (0.5%) cortisone-antibiotic combination (dexamethasone + netilmicin)
2. Carrying out the injection: a conjunctival motility test was done in all patients using cotton swabs to check the tissue mobilization and integrity before performing the sclerotomy. The injection was performed by keeping the tip of the oblique end of the 22-gauge needle up, beveled down, and making an initial tangential movement to the sclera, then adjusted towards the eye center until the needle was fully inserted. At this point, after pressing the button that causes the drug release in the vitreous cavity, the technique to pull the needle out follows the injection steps backwards, retracing the scleral tunnel created. In the end, the conjunctiva is released by sliding on the sclera and covering the entrance site with healthy conjunctiva. The procedure did not require the aid of an operating microscope or magnifying glasses. The surgeon was in an upright position, not seated, to be able to access the chosen site for the injection with the utmost comfort. It is better and desirable for the future to use the operating microscope associated with intraoperative OCT, to increase resolution and adequately assess preoperative and intraoperative conditions as well as immediate postoperative changes.
3. Medication: at the end of injection, the cotton swab is held to tamponade for a few seconds without applying pressure on the eye only in case of conjunctival bleeding; otherwise, we proceed with the instillation of two more drops of antibiotic-cortisone combination. Patients are then discharged unwrapped, without plastic cups or patches, and with the recommendation not to touch the eye and apply indomethacin eye drops 3 times a day for 7 days. Patients stay in observation for about 15–30 min after the injection.

### Optical coherence tomography

The sclerotomy wounds were imaged with the Heidelberg SPECTRALIS HRA (Heidelberg engineering) with an axial resolution of 18 μm, transverse resolution of 60 μm, and a scan speed of 2000 A-scans per s. The OCT device was used to scan across the scleral region, to traverse the incisions’ center, showing them in profile. The scans were evaluated for the presence and relative degree of wound gape along the path of the incisions. Using the Heidelberg SPECTRALIS HRA, we were able to acquire scans in the same location using specific software follow-up settings. For every analysis, at least 15 slices were taken. The OCT scans were collected on postoperative days 1, 8, and 15.

## Results

Thirty-three patients (33 eyes) were included in the study. The median age of patients was 72.7 years (range, 49 to 81 years). All patients were of Caucasian ethnicity, with 17 males and 16 females. The follow-up time was 15 days for all patients. The most common indication for surgery was diabetic macula edema (26 eyes). The details of patient data are presented in Table 1.

### Slit-lamp clinical examination

Following the intravitreal injection, the wounds were checked with a slit-lamp examination on day 1; none showed any evidence of leakage (e.g., conjunctival bleb formation) or scleral damage. Slit-lamp examination on postoperative day 1 revealed subconjunctival hemorrhage and conjunctival injection to varying levels (none to moderate) over the incision sites. The external scleral entry wound was visible for only 11 patients (3 in group 1, 5 in group 2, 3 in group 3) under slit-lamp examination. On postoperative days 8 and 15, all the subconjunctival hemorrhages were resolved, and the entry wound was not seen.

### Intraocular pressure

The median intraocular pressure (IOP) was 16.5 mmHg (no statistical difference was found in the three groups, *p* > 0.05) on postoperative day 1 and 18 mmHg (with no statistical difference in the three groups, range 11.5 to 24.5 *p* > 0.05) on postoperative day 15. We did not measure it on day 8 because the clinic protocol requires a measurement only on the first day and within the first month of the injection. Only 3 patients had ocular hypertension (23, 24, and 24.5 respectively as recorded values) resolved with modification of the topical ocular hypotensive therapy already in use, given that all three patients already had a diagnosis of glaucoma (POAG).

### Anterior segment optical coherence tomography of sclerotomies

On the OCT scans of the anterior segment, the results were different according to the selection group. In all 33 patients in the first postoperative day, it was not possible to detect any sign of conjunctival pathway with the ASOCT. Some signs of residual inflammation such as edema and conjunctival thickening were detected in patients who presented subconjunctival hemorrhage, which were resolved by the subsequent checks. In all 33 patients analyzed, the conjunctival pathway was not present and no signs of interruption of the conjunctival mucosa were visible in the OCT scans. This factor underlines how the displacement of the conjunctiva, in addition to the protection of the scleral pathway, confirms the minimal invasiveness of the applied technique.

The use of the intraoperative OCT and the operating microscope allows for a maximum spatial resolution for the analysis of the selected quadrant and a careful evaluation of the preoperative anatomical state of the conjunctiva and the sclera. It would also allow for the quantification of the displacement of the conjunctiva and the scleral thickness in relation to the anatomical characteristics of the subject (myopia, hyperopia …).

**Fig. 1:**
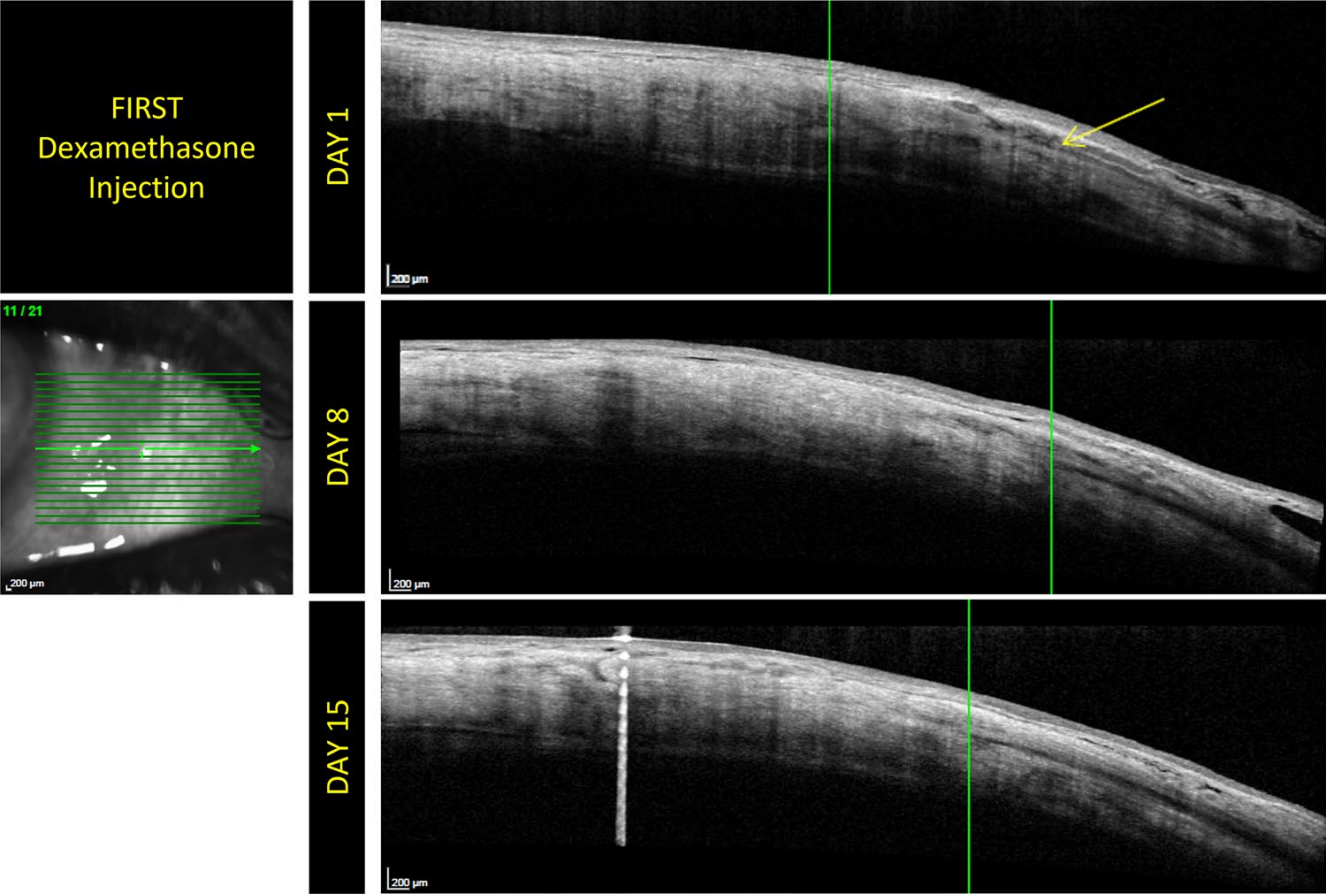
The OCT examination performed on a patient of group 1. Mild conjunctival edema is detectable on day 1. No conjunctival damage is detectable on days 8 and 15. It is possible to highlight the evolution of the scleral path. In the image corresponding to day 1, there is the residue of the first part of the scleral pathway which (yellow arrow) disappears in subsequent examinations. There are no edemas or other signs of residual inflammation

**Fig. 2:**
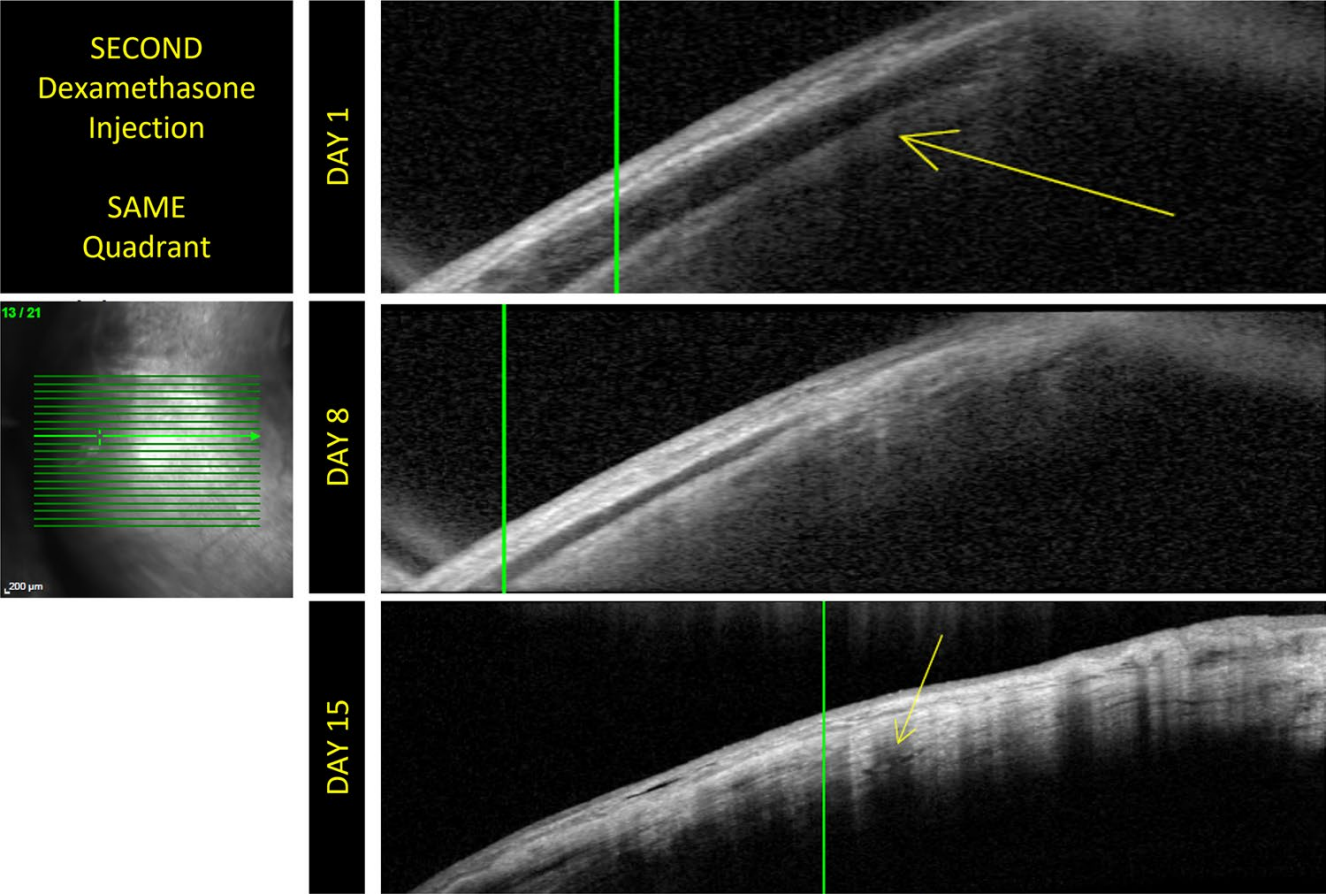
The OCT examinations performed on different patients belonging to group 2. Mild conjunctival edema is detectable on day 1. No conjunctival damage is detectable on days 8 and 15. It is possible to highlight the evolution of the scleral path. In this figure, the arrow in the scan corresponding to day 1 highlights the intrascleral edema surrounding the injector path. It is evident that the edematous area is reduced subsequently in the scan at 8 days and that only a scar remains present at 15 days

**Fig. 3:**
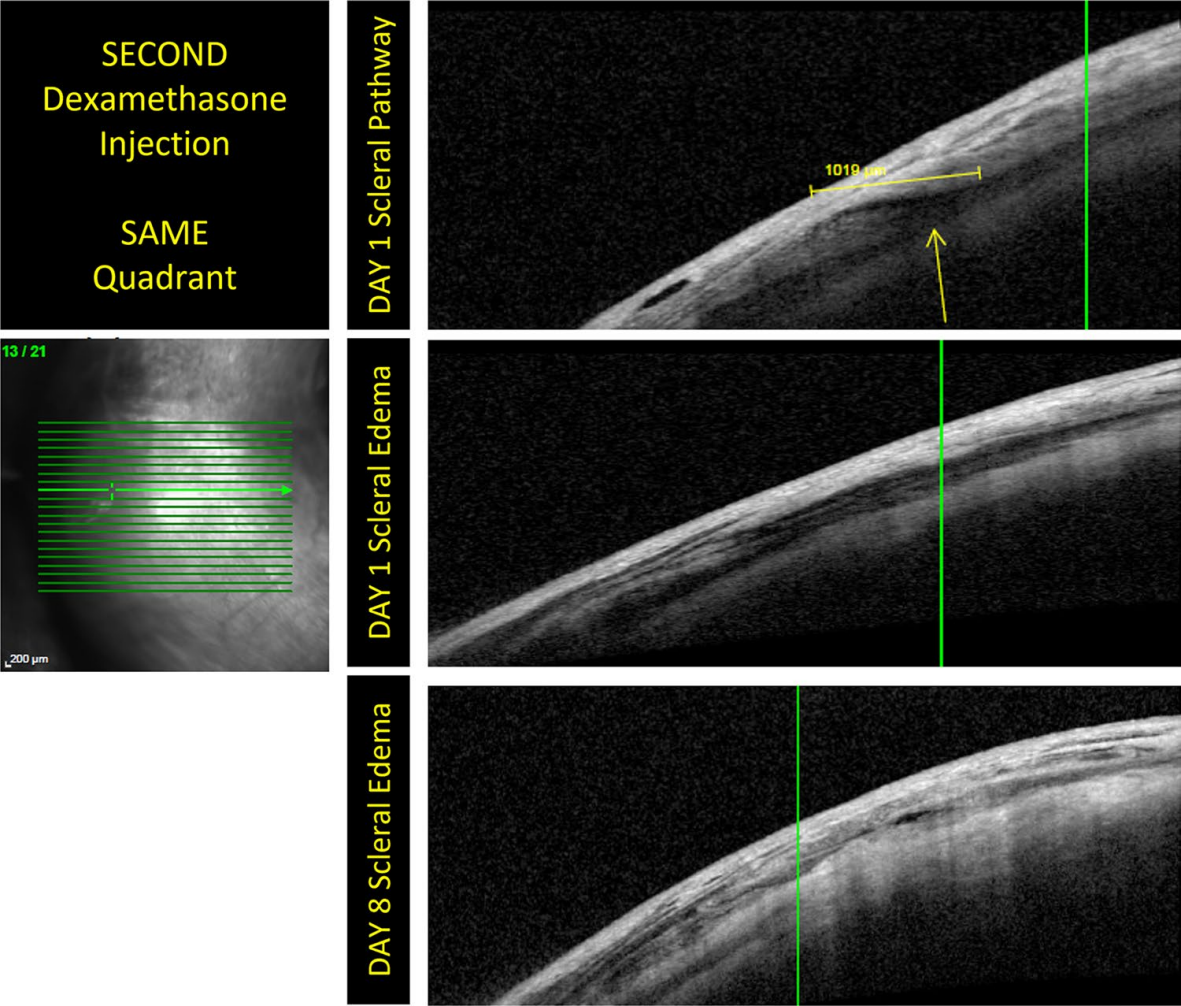
The OCT examinations performed on different patients belonging to group 2. Mild conjunctival edema is detectable on day 1. No conjunctival damage is detectable on days 8 and 15. It is possible to highlight the evolution of the scleral path. These images contain the scans of another patient and the evolution of the scleral anatomy following the injection. They show that injection site healing is slowed in patients who undergo a puncture at a site previously used for injection

**Fig. 4:**
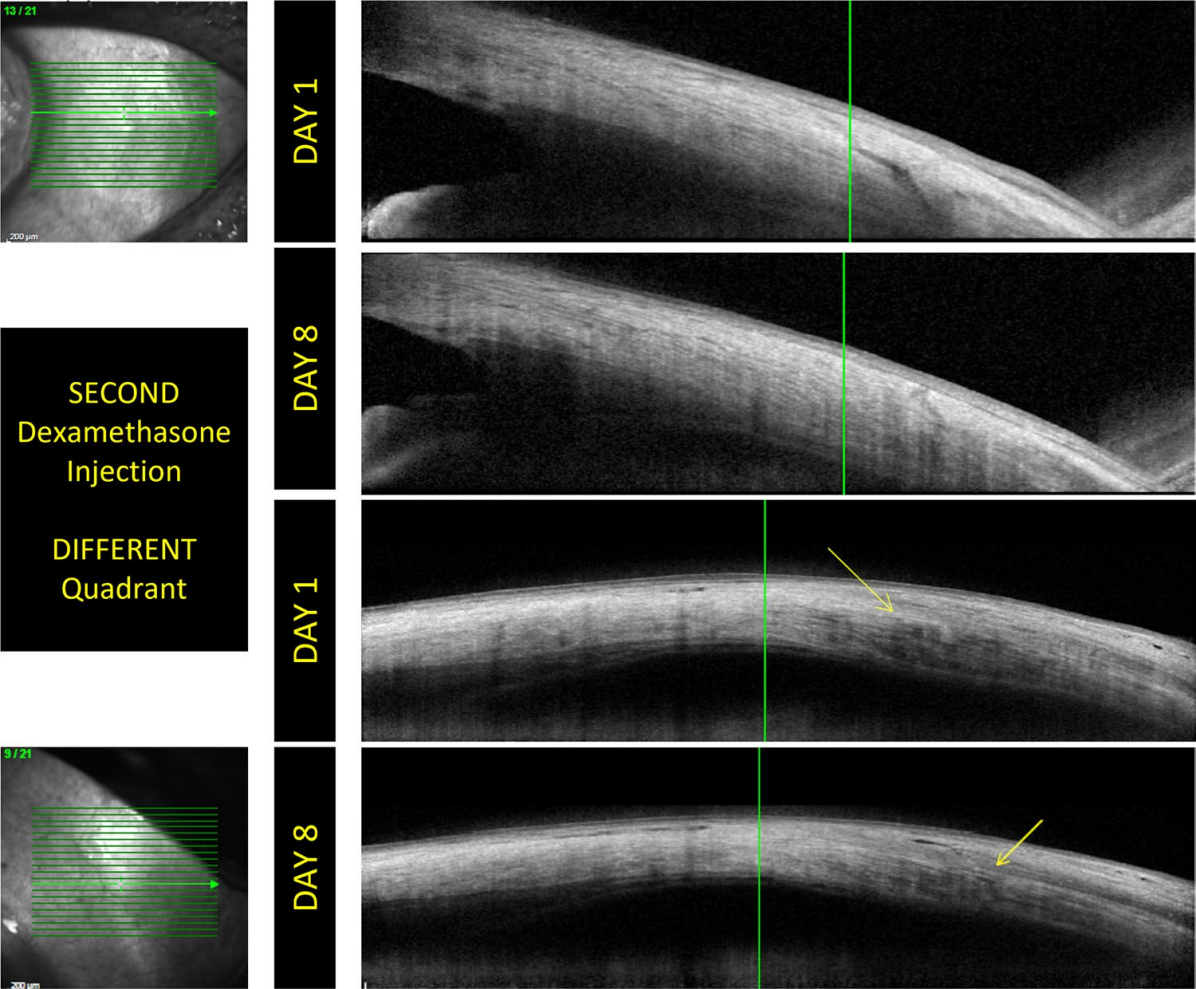
The images shows the evolution of the intrascleral pathway in two patients belonging to group 3. In these images, no conjunctival damage is detectable on days 8 and 15. In the first pair of acquisitions, it is shown that the intrascleral pathway disappears almost completely at 8 days. The evolution and resolution of intrascleral edema is evident in the second pair of images. The rapid evolution of scleral healing in group 3 patients highlights how the variation of the puncture site is useful for improving scleral *restitutio ad integrum*

In patients at the first dexamethasone injection, it was possible to identify and photograph the intrascleral pathway in 10 patients at the 1-day follow-up. There were no signs of intrascleral peri-wound edema or other detectable anatomical changes. In the 8-day follow-up, the sclera had returned to its *restitutio ad integrum* in 9 of the 10 patients; at 15 days, even in the last patient, there were no signs of scleral pathway (Fig. 1). In the patients of group 2, who performed a second or more puncture of dexamethasone in a quadrant already used previously, in all patients, the intrascleral pathway was recognizable on the first day of control; in 7 patients, this was accompanied by the presence of intrascleral edema with an accumulation of peri-wound fluid. In all patients, however, the deepest edge of the intrascleral tunnel was closed and any abnormal communication between the vitreous cavity and the subconjunctival space has been shown. At the 8-day checkup, 3 eyes still showed signs attributable to the intrascleral pathway accompanied by peri-wound edema; however, the disappearance of the signs of inflammation or sclerotomy was confirmed in all patients at the 15-day checkup, although a scar was evident in sclerotomy site, while in the other groups, we did not detect any scar visible to OCT (Figs. 2 and 3). In the patients of group 3, who performed a second or more dexamethasone puncture in a quadrant not used in the previous punctures, it was possible to identify the intrascleral pathway in 8 patients. There were no signs of intrascleral peri-wound edema or other anatomical changes in 9 patients of 10 belonging to this group as early as the first day. In the 8-day follow-up, the signs of scleral edema were absent in the single patient who presented them, and we find signs of the sclera in only one, the same, of the 8 patients (one of the patients suffering from glaucoma) (Fig. 4). At 15 days, there were no signs of scleral tunnel in all eyes analyzed.

All the results are summarized in Table 2.

**Table 2:** All the results investigated are summarized in this table

	Group 1	Group 2	Group 3
No. of patients	14	9	10
Age (average)	75	70	72
Slit-lamp examination			
External scleral wound	3	5	3
Day 1			
Sign of sclerotomy	10	9	8
Edema	0	7	1
Day 8			
Sign of sclerotomy	1	3	1
Edema	0	3	0
Day 15			
Sign of sclerotomy	0	0	0
Edema	0	0	0

### Other complications

Neither intraoperative nor postoperative complications were reported during the follow-up period. However, this study was not intended to evaluate the long-term outcome.

## Discussion

Literature regarding the correct procedure for intravitreal injections is incomplete. While for anti-VEGF drugs this is not a relevant issue given the relative ease of surgery; the thinness of the needle used, 30 G; and the low incidence of serious complications, it is not so true for the injection of dexamethasone. A marker of this difference is the incidence of endophthalmitis, found to be 6.92% higher [14] in steroid injections than in anti-VEGF drugs. Apart from the Allergan indications for the injector use [15], no real analyses of the protocols used pre-, post-, and intraoperative for the safety of the procedure are reported in literature.

The dexamethasone injector has a conformation and a diameter that are close to the diameters used in PPV: 22-G needle. It is significantly thicker and therefore more traumatizing to the scleral tissue than the devices employed for intravitreal injections of antibiotics or anti-VEGF therapy. The same precautions adopted in the surgical sclerotomy procedure should be used and studied with equal precision for the dexamethasone injection procedure and for hemorrhagic-exudative macular pathologies treated with anti-VEGF drugs. Certainly, the sclerotomy for PPV is maintained for a longer time than few seconds of stress of the intravitreal injection, but it should be remembered that the incidence of endophthalmitis following sutureless vitrectomy 25-G is 0.23% [16] and 0.84% [17] according to other studies. Therefore, it is difficult to argue the evidence that the endophthalmitis rate is consistent with sutureless 25-gauge vitrectomy. Why? The answer perhaps lies in wound construction. Kunimoto and Kaiser stated that no beveling of the incisions was performed in their series [16], while 73% of the endophthalmitis cases in Scott and associates’ series were from the straight incision [17]. Moreover, there have also been reports of higher hypotony rates associated with sutureless vitrectomy using straight incisions [18][18][18]. This may be attributable to wound leak, which would increase the risk of endophthalmitis.

Applying the technique recognized as safest for sclerotomies during vitrectomy, the injection needle should first be placed tangentially to the inner sclera and then perpendicular to the center of the eye globe, which creates a valved tunnel undergoing compressing forces owing to the configuration of the scleral fibers. This valved tunnel combined with the surface tension between the outer scleral walls facilitates sealing of the wound. Oblique or angled incisions are believed to help decrease or prevent wound leakage, by having an internal lip that presses against the outer lip through IOP, thereby helping to close the wound [21][21][21]. The importance of creating oblique incisions as opposed to straight incisions was highlighted in several recent in vitro studies where angled incisions were superior macroscopically and histopathologically, and with anterior segment OCT [24][24].

Haller et al. [3] analyzed in their study the safety of using the dexamethasone injector for intraocular dexamethasone implant. Various parameters were evaluated, including IOP and BVCA as well as the cataract onset following one or more injections. The scleral damage and its evolution were not investigated. Only in a letter to the authors, from Gonzaga Garay-Aramburu and Javier Cabrerizo [26], they reported their assessment of the damage by OCT, confirming how at 1 day the scleral tunnel created by the needle could be visualized and how the closure of the same occurred within the first 8 days. Haller’s data for safety in patients with venous thrombosis were also confirmed by Boyer et al. [5] for patients with diabetic macular edema, but, even in this case, the evaluation of scleral safety had not been done.

The intensive use of this type of injector has led many patients to receive more than 2–3 injections of slow-release dexamethasone during the same year. Over time, this treatment regimen can lead to several sclerotomies, much higher in number than those performed during even multiple vitrectomies. It remains challenging to analyze the status of conjunctiva, how the sclera reacts to multiple perforations, and how the healing process led to a true *restitutio ad integrum* for both over time, but it may be the purpose of future studies. Currently, this study is the first that evaluates the differences in ASOCT imaging of conjunctiva status and the changes in scleral morphology after multiple injections in the same quadrant compared to single or repeated injections with a quadrant variation. The limit that the authors recognize in this first study is given by the number of patients analyzed. This number corresponds to the total number of patients undergoing the surgical procedure in the analysis time of the applied methodology. The morphological correlations found by OCT examination are and will always be independent of the number of patients enrolled in this study.

From data presented in this study, it seems to be clear how injecting dexamethasone in a different site from the previous one results in faster healing and less invasiveness (e.g., absence of intrascleral edema). On the other hand, repetition of several injections in the same quadrant exposes to slower healing, with greater infectious disease risk, and a more aggressive inflammatory response with consequent thinning of the scleral wall.

In any case, the conjunctival displacement has proven effective in protecting the complete reparative phase of the sclera, given that the total and rapid closure of this isolates the subconjunctival space from the outside. This kind of precaution should be adopted in any case. The use of cotton swabs before injection helps to choose the site with the greatest conjunctival mobility; where there will be the best sliding, there will be the maximum protection.

It is therefore advisable to change the quadrant in which the injection is to be made for each new treatment. Classically, the inferotemporal quadrant represents the easiest choice due to the width and absence of large conjunctival vessels. Even the lower sectors, for the same reasons, are often chosen by surgeons as favorites. The upper and nasal sectors are usually avoided, but by sliding the conjunctiva, the vascular damage and the risk of conjunctival hemorrhage can be averted and the use of the blepharostat, accompanied by the recommendation to the patient to move the eye in the opposite direction to the chosen site, leads to sufficient exposure and safety.

Injection of dexamethasone using a preloaded device remains a highly safe procedure, even for patients with complications or previous surgery. Any single anatomical features need to be carefully evaluated and considered especially in a simple and repetitive surgical technique such as intravitreal injection: this caution is the basis for the prevention of those rare, albeit present, complications, such as the infectious risk of endophthalmitis, which must be avoided in every possible way. This surgical technique, which reduces the risk of ocular complications, allows for clinical needs with or without associated pathologies to be able to carry out more injections of dexamethasone in shorter times without increasing the risk of ocular complications but rather preventing them.

In the case of repeated injections on the same eye, careful choice based on the history of the injection site can lead to improvement in terms of speed of postoperative recovery. It would therefore be important that data regarding the chosen quadrant be marked on the medical report, so that it can be consulted at the subsequent administration. ASOCT is a very important and increasingly accurate technology [27], capable of quantifying and following the correct healing of sclerotomy. Its implementation can be recommended in all patients who have performed numerous intravitreal injections, those who are predisposed to complications, myopic and hyperopic patients of various degrees, glaucomatous, mostly if decompensated.

## Conclusions

Given the many patients undergoing and the wider range of error, especially for simple, repetitive surgical procedures, it may be appropriate that surgical protocols be studied and shared in detail. In case of intravitreal injections of slow-release dexamethasone, the technique involving displacement of the conjunctiva and a beveled or angled sclerotomy after a careful selection of the injection site, changing injection quadrant involved with each administration, reduces the number of days required for scleral tunnel obliteration, thereby protecting the patient from vitreous reflux and related complications, especially endophthalmitis or panuveitis or posterior uveitis. The improvement of the technique will be achieved if, in the future, there will be the widespread possibility of using the operating microscope and intraoperative OCT for the execution of injections of dexamethasone implants.
